# A Timing Estimation Method Based-on Skewness Analysis in Vehicular Wireless Networks

**DOI:** 10.3390/s151128942

**Published:** 2015-11-13

**Authors:** Xuerong Cui, Juan Li, Chunlei Wu, Jian-Hang Liu

**Affiliations:** Department of Computer and Communication Engineering, China University of Petroleum (East China), Qingdao 266580, China; E-Mails: lijuanlijuan@sina.com (J.L.); wuchunlei@upc.edu.cn (C.W.); liujianhang@upc.edu.cn (J.-H.L.)

**Keywords:** vehicular wireless networks, vehicle positioning, timing estimation, dynamic threshold, positioning sensor, IEEE 802.11p, skewness analysis

## Abstract

Vehicle positioning technology has drawn more and more attention in vehicular wireless networks to reduce transportation time and traffic accidents. Nowadays, global navigation satellite systems (GNSS) are widely used in land vehicle positioning, but most of them are lack precision and reliability in situations where their signals are blocked. Positioning systems base-on short range wireless communication are another effective way that can be used in vehicle positioning or vehicle ranging. IEEE 802.11p is a new real-time short range wireless communication standard for vehicles, so a new method is proposed to estimate the time delay or ranges between vehicles based on the IEEE 802.11p standard which includes three main steps: cross-correlation between the received signal and the short preamble, summing up the correlated results in groups, and finding the maximum peak using a dynamic threshold based on the skewness analysis. With the range between each vehicle or road-side infrastructure, the position of neighboring vehicles can be estimated correctly. Simulation results were presented in the International Telecommunications Union (ITU) vehicular multipath channel, which show that the proposed method provides better precision than some well-known timing estimation techniques, especially in low signal to noise ratio (SNR) environments.

## 1. Introduction

Currently, vehicle collision accident rates remain high and cause great damages every year. The World Health Organization (WHO) has indicated that globally road traffic injuries are the eighth leading cause of death [1]. More than a million people die each year on the World’s roads and current trends suggest that by 2030 road traffic deaths will become the fifth leading cause of death unless more action is taken. One effective action is the application of cooperative collision avoidance (CCA), by which vehicles can predict potential hazards and remind the drivers in advance about hazards by exchanging real-time information such as location, direction, speed, acceleration and so on. For a CCA system, the vehicle’s location is one of the most important parameters to be estimated. If drivers could receive a collision warning 0.5 s before an incident, 60% of collisions could be avoided, but how to accurately and reliably estimate the vehicle’s location is still a challenge. Without an accurate position estimation, the CCA systems cannot produce timely alerts about potential dangers ahead or may provide some unwanted warnings when there is no danger ahead.

Currently, global navigation satellite systems (GNSS) such as the US global positioning system (GPS) and the BeiDou satellite positioning system (BDS) of China have been widely used in vehicular wireless networks to reduce transportation time and traffic accidents. If the satellite signals are blocked in a non-line of sight (NLOS) environment, such as in urban areas where tall buildings or overpass bridges or dense foliage may block the view of the sky they will fail to obtain accurate position estimations. GNSS must therefore be integrated with some other positioning technique such as short range wireless position, Inertial Navigation System (INS), digital maps, and so on. The Chinese Area Positioning System (CAPS) is a regional navigation satellite system. In [2] ultra-wide-band (UWB) pseudo-satellite signals were used in the CAPS, and the integrated method could provide better performance than CAPS itself. A three layer framework was presented in [3] to monitor the performance of real-time relative positioning in vehicular wireless networks that support CCA applications. The IEEE 802.11p [4] standard is just employed to translate the GPS real-time kinematic (RTK) information. Unfortunately, these systems also perform poorly in NLOS environments.

Nowadays, wireless mobile communications systems or cellular networks [5] are widely used in many areas, including in vehicular wireless networks, but they cannot be used for positioning, because they have no real-time communication ability. This is because each message will be delivered through different cellular base stations, which will introduce insufferable time delays compared with the rapid velocity of vehicles. In addition, the positioning precision of cellular signals is about several hundred meters so it can't meet the demands of CCA systems. Traditional wireless sensor networks [6] or indoor positioning methods [7,8] are not applicable in vehicle environments, because of the severe Doppler frequency shift caused by the high velocity of vehicle movement. Fortunately, IEEE 802.11p was approved in 2010 to add wireless access in vehicular environments, which made some amendments to the IEEE 802.11 in the physical (PHY) and media access control (MAC) layers to achieve a robust connection and a fast setup, such as the longer symbol duration, guard time and FFT period.

In order to improve the timing precision for vehicular nodes, a new method with a dynamic threshold using the skewness analysis of IEEE 802.11p short preamble is proposed in this paper. Against this background, the OFDM preamble structure is analyzed in Section 2. Section 3 introduces the related work on timing estimation, and the proposed method is put forward in Section 4. Simulation setup and result analysis are discussed in Section 5, and finally Section 6 concludes the paper. The main contributions of this paper include:

(1) A new cross-correlation, summing up and skewness analysis (denoted CSS) method is proposed which can be used to estimate the time offset in positioning sensors of vehicular wireless networks.
(2) The CSS method provides better precision than some well-known time estimation techniques, especially in low signal to noise ratio (SNR), and multi-path environments, which is very important for vehicular wireless networks.
(3) The CSS method can also be used in other wireless communications which use OFDM with repeated preamble symbols, such as IEEE 802.11a.

## 2. OFDM Preamble Structure

IEEE 802.11p specifies the extensions to the PHY and MAC layers of IEEE 802.11 for wireless local area networks providing wireless communications while in a vehicular environment, e.g., at high speeds of up to 200 km/h, multi-path fading and long range of range of 1000 m. In IEEE 802.11p, the same OFDM modulation technique and preamble structure as in IEEE 802.11a [9] are used to mitigate the effects of fading, but the larger symbol duration is specified, which results in a longer guard interval (GI) and can provide more tolerance to greater delay spreads and inter-symbol interference (ISI). Thus, IEEE 802.11p can deal with the severe Doppler frequency shifts, and rapidly changing multi-path conditions. 

### 2.1. Modulation Technique

With the modulation technique of OFDM, the inverse fast Fourier transform (IFFT) is used to generate the OFDM symbols. In each OFDM symbol, there are multiple orthogonal subcarriers. In IEEE 802.11p, the channel bandwidth of each subcarrier is 10 MHz and the carrier frequency is 5.9 GHz. Therefore, the frequency spacing between each subcarrier is ${∆}_{F}$ = 10/64 = 0.15625 MHz, which is half of the channel bandwidth of IEEE 802.11a. The duration of IFFT (*T*_FFT_) in IEEE 802.11p is 6.4 μs, which is twice that of IEEE 802.11a. The duration of GI is 1.6 μs.

### 2.2. Preamble Symbols

**Figure 1:**
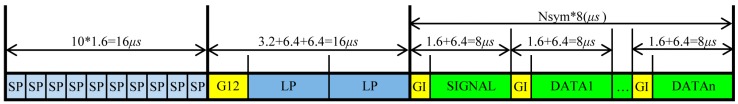
Format of IEEE802.11p PHY layer.

The PHY layer format of IEEE802.11p is shown in Figure 1. The first two part are the preamble fields, which are divided into two parts, 10 short preamble symbols and two long preamble symbols. In the frequency-domain, each short preamble symbol consists of 12 nonzero subcarriers located at positions ±(4, 8, 12, 16, 20, and 24) which gives:

(1) $$S_{-26,26}=\sqrt{13/6}\left\{0,0,+1+j,0,0,0,-1-j,0,0,0,+1+j,0,0,0,-1-j,0,0,0,-1-j,0,0,0,+1+j,0,0,0,0,0,0,0,-1-j,0,0,0,-1-j,0,0,0,+1+j,0,0,0,+1+j,0,0,0,+1+j,0,0,0,+1+j,0,0,\;\right\}$$

The OFDM symbol is defined as:

(2) $$s\left(t\right)=w\left(t\right)\overset{k=N/2}{\underset{k=-N/2}{\sum }}S_{k}exp\left(j2\pi k{∆}_{F}t\right)$$

where *N* = 52 and w(t) is the window function. Because there are only 12 nonzero subcarriers, the factor $\sqrt{13/6}$ is used to normalize the average power of the OFDM symbol. Because subcarriers *s* has nonzero values only for indices that are a multiple of 4, the short preamble symbol duration is *T*_FFT_/4 = 1.6 μs. Therefore, the duration of the ten short preamble symbols is 10 × 1.6 = 16 μs. 

There are 52 nonzero subcarriers and 12 null subcarriers with a duration of 6.4 μs in each long preamble symbol. Between short preamble and long preamble, there are two guard interval symbols (G12) with the duration of 1.6 μs. Therefore, totally, the duration of the preamble is 10 × 1.6 + 2 × 1.6 +2× 6.4 = 32 μs.

The short preamble symbols are typically used for coarse timing estimation and the long preamble symbols are used for fine timing estimation and frequency shift estimation. The former is critical to the success of the fine estimation so it is considered in this paper.

## 3. Related Work

Most of the wireless positioning methods are based on ranging or timing delay estimation from the base station to the target positioning sensor (the vehicular node to be positioned), for example time of arrival (TOA) [10], time difference of arrival (TDOA) and received signal strength (RSS) [11,12]. However, there is a very challenging problem for ranging estimation due to the severe multi-path, reflection, and inter-symbol interference environments encountered [13,14]. Therefore, the ranging or timing delay estimation problem has been extensively studied. Ranging or positioning methods in the traditional application sense have been studied more extensively [15,16,17], but in the vehicle environment less so [18]. Because orthogonal frequency division multiplexing (OFDM) is used in the IEEE802.11p standards, we focus on some timing methods for OFDM.

### 3.1. SC Method

Schmidl and Cox developed the well-known timing estimation method (denoted as SC) [19] based on OFDM, which is recommended in the IEEE802.11a standard [20]. However, the timing metric will reach a plateau which leads to uncertainty in the timing estimation. In the SC method [19], the preamble symbols can be descripted as the pattern of [GI A A] where GI is the guard interval symbols which are followed by two groups of identical symbols. The metric for timing estimation in SC method is given by:

(3) $$M\left(d\right)=\frac{{\left|P\left(d\right)\right|}^{2}}{R^{2}\left(d\right)}$$

where *P*(*d*) is the auto-correlation of the received samples and *R*(*d*) is the energy of the received samples*,* given by:

(4) $$P\left(d\right)=\overset{L-1}{\underset{m=0}{\sum }}r^{*}\left(d+m\right)\cdot r\left(d+m+L\right)$$

(5) $$R\left(d\right)=\overset{L-1}{\underset{m=0}{\sum }}{\left|r\left(d+m+L\right)\right|}^{2}$$

where *L* is length of the received samples, *d* is the time index of the received samples and * is the complex conjugate. 

**Figure 2:**
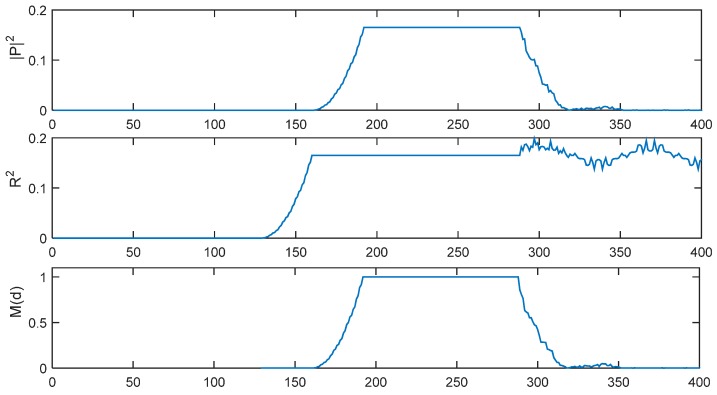
|*P*(*d*)|^2^, *R^2^*(*d*) and *M*(*d*) of SC method with *L* = 32 in ideal environment.

Figure 2 presents |*P*(*d*)|^2^, *R*^2^(*d*) and *M*(*d*) with *L* = 32 in an ideal channel with a time delay of 191 samples. For the IEEE802.11p protocol, there are 160 short preamble samples, so the first 160 − 2*L* = 96 samples are considered to be the GI. The timing metric *M*(*d*) forms a plateau which has a length equaling to the length of the GI (*N*_g_ = 96 samples) minus the length of channel delay spread. In an AWGN channel, there will be no delay spread, so the length of the plateau is 96 samples. 

### 3.2. RMB Method

**Figure 3:**
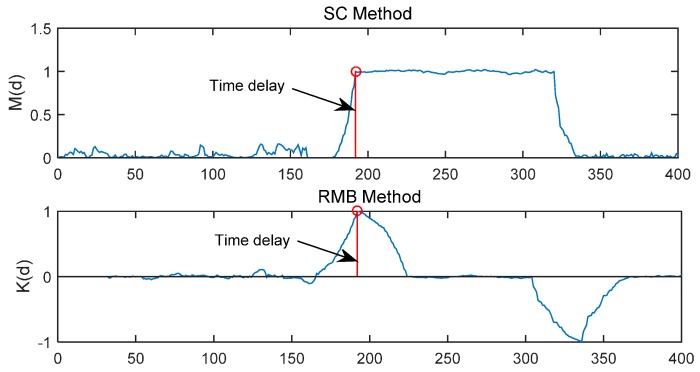
*M*(*d*) of SC method and *K*(*d*) of RMB method.

In [21], Reddy, Mahanta, and Bora have modified the timing metric to reduce the plateau, however the single peak will be affected by noises, so the timing estimation is still uncertain. The new timing metric *K*(*d*) is shown as:

(6) K(d)=M(d)−M(d−L)

where *K*(*d*) will reach the maximum value at the start of training sequence and fall subsequently, thus, there is no plateau. As shown in Figure 3, the *M*(*d*) of SC method and the K(d) of RMB method are simulated in an AWGN channel with *E*_b_/*N*_0_ (the energy per bit to noise power spectral density ratio) = 30 dB, time delay = 191 samples and *L* = 16.

### 3.3. MathWorks Method

The SC and RMB methods use auto-correlation, but in the method of Matlab [22], MathWorks employs cross-correlation between the received signal and the short preamble given in Equation (2). The timing metric *M*(d) is the same as the SC method in Equation (3), but *P*(d) is defined as:

(7) $$P\left(d\right)=\overset{L-1}{\underset{m=0}{\sum }}r\left(d+m\right)\cdot t^{*}\left(1+m\right)$$

where *t*(1 + *m*) is the short preamble sequence, *i.e*., the reference template, and *R*(d) is the received energy defined as:

(8) $$R\left(d\right)=\overset{T-1}{\underset{m=0}{\sum }}{\left|r\left(d+m\right)\right|}^{2}\;\;$$

with length *T* = 16. Because of the repeat of the short preamble samples, there will be nine peaks in the timing metric *M*(d). In the MathWorks method, during the timing period, the value of 60% × maximum of *M*(d) is used as a threshold to determine the time offset. If the peaks exceeding the threshold are located in the spacing of 16 times (16, 32, 48,…, 128) and the count of these peaks is more than 5, then the location of the first peak ($L_{first}$) is deemed as the peak of the first path, and the timing delay (${\hat{D}}_{\text{est}}$) is estimated as:

(9) $${\hat{D}}_{\text{est}}=\underset{\;}{L_{first}-\left(\frac{L}{2}+1\right)}\;$$

**Figure 4:**
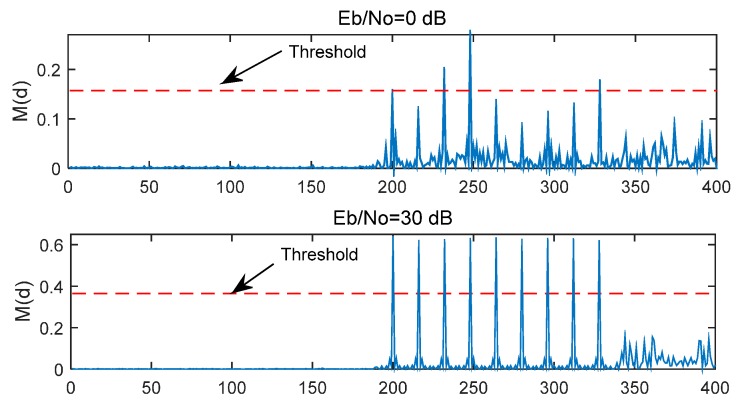
*M*(*d*) of MathWorks method in different *E*_b_/*N*_0_.

Two timing metric examples with *E*_b_/*N*_0_ = 0 dB and 30 dB are simulated in Figure 4 and the time delay is 191 samples. In these cases, the first peak should be located at $L_{first}=\;$191 + 16/2 + 1 = 200th sample. As shown in Figure 4, When *E*_b_/*N*_0_ = 30 dB, the nine peaks are very sharp and the first peak appears at the 200th sample. When *E*_b_/*N*_0_ = 30 dB, all nine peaks exceed the threshold, but when *E*_b_/*N*_0_ = 0 dB, there are only two peaks exceeding the threshold, so the timing offset cannot be estimated accurately. In order to solve this problem, the following two steps are put forward in the following Section. 

### 3.4. Liu et al. Method

**Figure 5:**
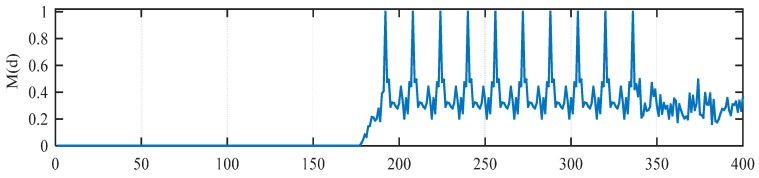
*M*(*d*) of Liu method in ideal environment.

Auto-correlation based methods, such as the SC method or RMB method, are immune to carrier frequency offset (CFO), but their timing metric is not sharp enough. On the other hand, a cross-correlation based method such as the MathWorks method has sharp timing metrics, but its performance is sensitive to CFO. In [23], Liu *et al*. proposed a new timing method which used both auto-correlation and cross-correlation, so this method has the advantages of both approaches. Figure 5 shows the timing metric *M*(*d*) for the Liu *et al*. method in an ideal channel with a time delay of 191 samples. Because of the 10 identical short preamble symbols in IEEE 802.11p, there will be 10 peaks spaced 16 samples apart in an ideal environment, which may lead to uncertainty in the timing estimation just like the MathWorks method.

## 4. Proposed Timing Estimation Method

In this paper, we will pay attention to the correlation receive, that is to say, the received signal is correlated with the reference template (the short preamble symbols), which is given by:

(10) $$r_{xs}\left(\tau_{d}\right)=\frac{1}{T_{0}}\int_{T_{0}}p\left(t\right)r\left(t-\tau_{d}\right)dt$$

where *T_0_* is the correlation duration, *p*(*t*) is the reference template, the short preamble symbols, and *r*(*t*) is the received signal.

The receiver can estimate the time offset according to the output samples from the correlator. Then, the range between the transmitter and receiver nodes can be calculated according to this estimated time offset. Several techniques have been developed for OFDM-based timing estimation. If the reference template signal is selected from the received signal, this method is called auto-correlation, otherwise it is a cross-correlation. The main steps of the proposed method contain cross-correlation, summing-up, and skewness-analysis, so it is denoted as the cross-correlation, summing-up, and skewness-analysis (CSS) method.

### 4.1. Cross-Correlation

The correlation is the same as in the MathWorks method which includes the Equations (7), (8), and (3). As shown in Figure 4, in an ideal environment, there will be nine peaks in the timing metric *M*(*d*) and the spacing between each adjacent desired peak is 16 samples. However, if *E*_b_/*N*_0_ is low, the noise can affect the number of peaks that exceed a specific threshold, and it is difficult to determine the optimal threshold value, this is why the performance of the MathWorks method is relatively low.

### 4.2. Summing-Up

The summing-up of nine correlated samples *M*(*d*) with a pace of *T* = 16 samples apart are considered, as shown in Equation (11):

(11) $$G\left(t\right)=\overset{9}{\underset{d=1}{\sum }}M\left(t+\left(d-1\right)\text{*}L\right)$$

and the sample index (${\hat{D}}_{\text{est}}$) responding to the time delay is obtained as:

(12) $${\hat{D}}_{\text{est}}=\underset{\;}{\text{argmin}\left(t|G\left[t\right]\geq \xi *\max\left(G\left(t\right)\right)\right)-\left(\frac{T}{2}+1\right)}\;$$

where ξ is a threshold between 0 and 1.

**Figure 6:**
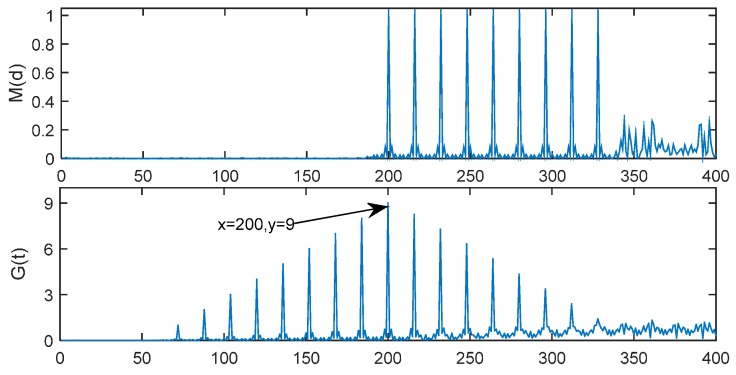
*M*(*d*) and *G*(*t*) in ideal environment.

In ideal environment with no noise, the new timing metric *G*(*t*) is simulated in Figure 6 with time delay = 191 samples. Now, there are 17 symmetrical peaks and the location of the maximum peak is just the index to be found, which should be located at the 191 + 16/2 + 1 = 200th sample.

**Figure 7:**
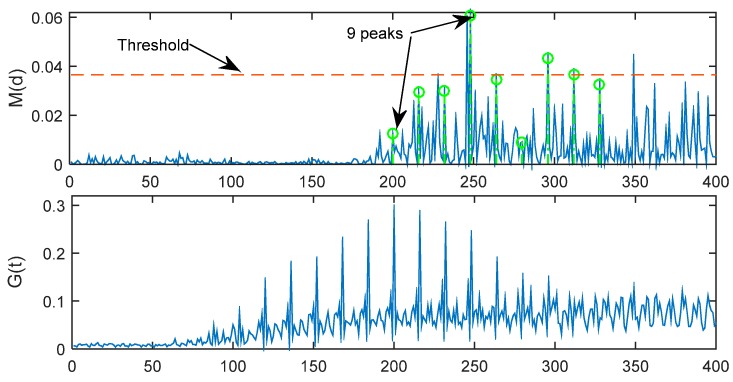
*M*(*d*) and *G*(*t*) with *E*_b_/*N*_0_ = −5 dB.

As *E*_b_/*N*_0_ decreases, the timing metric *M*(*d*) of MathWorks method will become more and more hard to be identified, but the new timing metric *G*(*t*) is better. As shown in Figure 7, when *E*_b_/*N*_0_ = −5 dB, the 9 peaks of *M*(*d*) are very hard to be identified, but the maximum of *G*(*t*) is very clear, and is located at 200th sample, and then can be estimated correctly.

**Figure 8:**
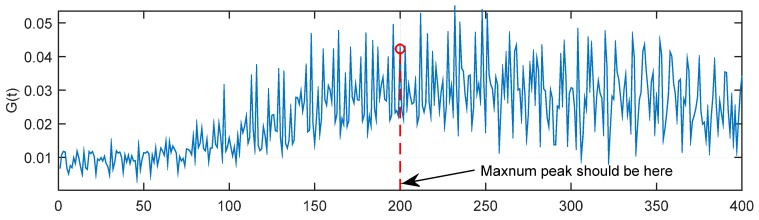
*G*(*t*) with *E*_b_/*N*_0_ = −10 dB.

However, as *E*_b_/*N*_0_ decreases more and more, the *G*(*t*) will become more difficult to identify by a fixed threshold. As shown in Figure 8, when *E*_b_/*N*_0_ = −10 dB, the maximum of *G*(*t*) is very hard to locate in the 200th sample. That is to say, the maximum of *G*(*t*) is not the proper sample index. At the same time, it is very hard to determine the threshold ξ to find the proper index. Therefore, in the following part of this Section, a dynamic threshold method is put forward.

### 4.3. Skewness-Analysis and Threshold

In order to improve the precision in low *E*_b_/*N*_0_, a dynamic threshold using the skewness analysis of IEEE 802.11p short preamble is proposed. In this Section, the skewness and standard deviation (STD) of the *G*(*t*) are analyzed. Then, in order to determine the best threshold (*ξ_best_*), the relationship between estimation error and the skewness of *G*(*t*) was investigated. Finally the relationship between skewness and dynamic threshold is set up to estimate TOA.

#### 4.3.1 Statistical Characteristics of the Summing-Up Samples

(1) Standard Deviation

STD is used to measure how much variation there is from the average of *G*(*t*), which is given by:

(13) $$\delta =\sqrt{\frac{\sum_{i=1}^{N_{b}}{\left(x_{i}-\bar{x}\right)}^{2}}{\left(N_{b}-1\right)}}$$

where *N*_b_ is the number of *G*(*t*) samples and *x*_i_ is the *i*th value of *G*(*t*).

(2) Skewness

The skewness of *G*(*t*) is given by:

(14) $$S=\frac{1}{\left(N_{b}-1\right)\delta^{3}}\overset{N_{b}}{\underset{i=1}{\sum }}{\left(x_{i}-\bar{x}\right)}^{3}$$

where $\bar{x}$ is the mean of *G*(*t*), and *δ* is the standard deviation of *G*(*t*). If *G*(*t*) is a normal distribution, *S* will be zero. If *S* < 0, *G*(*t*) will skew left which indicates that the left tail is longer than the right one, while if *S* > 0, *G*(*t*) will skew right and the left tail is shorter than the right one. As the *E*_b_/*N*_0_ increases, *S* will tend to increase. If there are no signals (or *E*_b_/*N*_0_ is too low), *S* will be zero.

**Figure 9:**
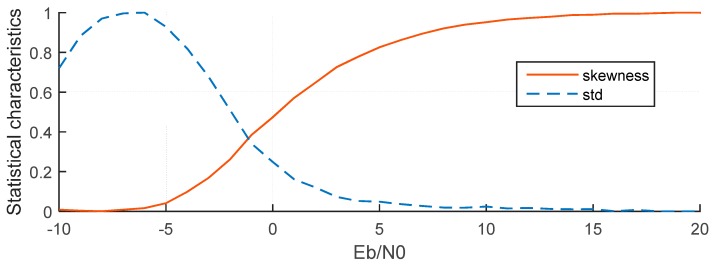
Two normalized statistical parameters change with *E*_b_/*N*_0_.

In order to examine the characteristics of the two statistical parameters, the ITU channel model is employed. For each *E*_b_/*N*_0_ from −10 dB to 20 dB, 2000 channel realizations were generated. The average results of STD and skewness are normalized and shown in Figure 9 which showa that the skewness increases as the *E*_b_/*N*_0_ increases but the STD increases at first and then decreases as the *E*_b_/*N*_0_ increases. Therefore, skewness is a monotonic function for a large range of *E*_b_/*N*_0_ values, and it is more suitable for TOA estimation than STD, because it can better reflect changes in *E*_b_/*N*_0_.

#### 4.3.2. Relationship between Estimation Error, Skewness and Threshold

In order to determine the best threshold (*ξ_best_*) based on skewness, the relationship between estimation error, skewness and threshold was investigated. 2000 ITU channel realizations with each *E*_b_/*N*_0_ = {−10, 5,…, 20} dB were simulated. With each channel realizations, the thresholds of {0.05, 0.10, 0.15, 0.20,…,1.0} are compared with *G*(*t*) to find the first threshold crossing sample index, as shown in Equation (12). 

**Figure 10:**
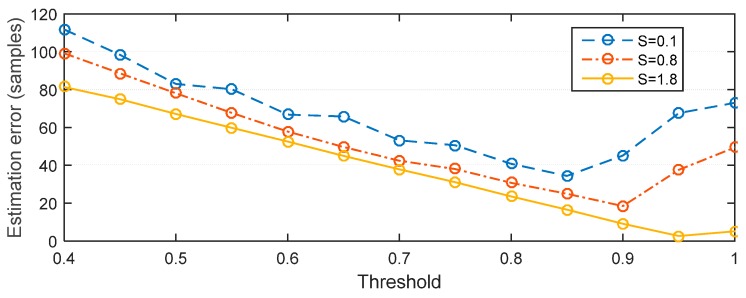
Relationship between MAE, threshold and skewness.

To illustrate the results, Figure 10 shows the estimation error for *S* = {0.1, 0.8, 1.8} in the ITU channels respectively. The relationship is that for a specific *S*, as the thresholds increasing, the estimation error decreases at first and then increases. For example, the threshold responding to the lowest errors for *S* = {0.1, 0.8, 1.8} are 0.85, 0.9 and 0.95. Thus, the threshold with respect to the minimum estimation error is selected as the best threshold *ξ_best_*.

#### 4.3.3. Relationship between Skewness and Threshold

A polynomial with degree 3 was fitted to the best threshold *ξ_best_* for each value of *S* by using the method of least-squares where *S* is the *x*-coordinate and *ξ_best_* is the *y*-coordinate. The result of the fitting will generate the coefficients of the polynomial with the minimized summed square of residuals. The *i*th residual *r_i_* for the *i*th pair of (*S*, *ξ_best_*) is defined as:

(15) $$r_{i}=y_{i}-{\hat{y}}_{i}$$

where *y_i_* is the best threshold and *ŷ_i_* is the fitted threshold value for the *i*th *S*, so the summed square of residuals *S*_S_ is given by:

(16) $$S_{s}=\overset{n}{\underset{i=1}{\sum }}r_{i}^{2}=\overset{n}{\underset{i=1}{\sum }}{\left(y_{i}-{\hat{y}}_{i}\right)}^{2}$$

where *n* is the number of (*S*, *ξ_best_*).

**Figure 11:**
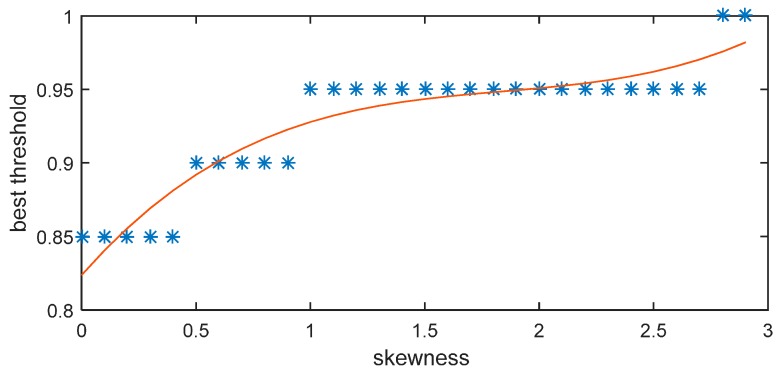
Relationship between skewness and threshold.

The least-squares fitting result is shown in Equation (17) and Figure 11:

*ξ* = 0.016085*S*^3^−0.088866*S*^2^ + 0.17697*S* + 0.82368 (17)

## 5. Simulation Setup and Result Analysis

### 5.1. Simulation Setup 

#### 5.1.1. System Model

Because vehicles are typically in motion, there will be a Doppler shift given by:

(18) $$F_{d}=v*f_{c}/c$$

where *v* is the relative vehicle speed which is typically set to *v* = 100 km/h, *f*_c_ = 5.9 GHz is the center frequency of the transmitted signal, and *c* is the speed of light. 

**Figure 12:**
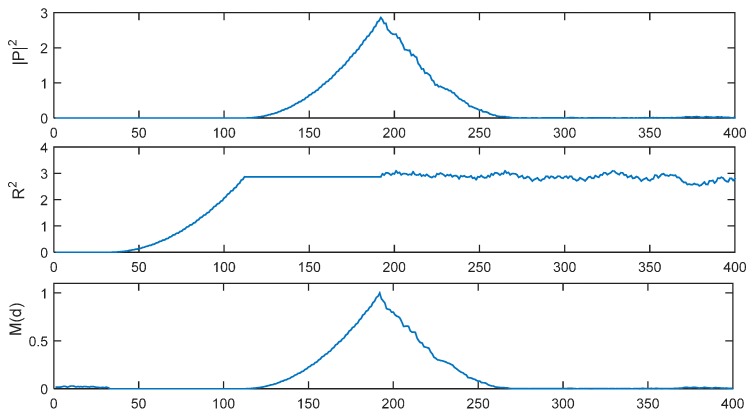
|*P*(*d*)|^2^, *R^2^*(*d*) and *M*(*d*) of SC-80 method in ideal environment.

The SC method with *L* = 32 has a plateau as shown in Figure 2, so the timing estimation is difficult. To mitigate this problem, *N*_g_ should equal to 0, so *L* = 80 is used (denoted by SC-80). The timing metric of SC-80 is presented in Figure 12 for an ideal environment with a time delay of 191 samples, and this indicates that there is no plateau. The other parameters for the simulation are shown in Table 1.

**Table 1:** Parameters for the simulation.

Parameters	Value
Modulation mode	BPSK
Number of subcarriers	52
Symbol duration	8 μs
Guard time	1.6 μs
FFT period	6.4 μs
Preamble duration	32 μs
Subcarrier spacing	0.15625 MHz
Vehicle speed	100 km/h
Channel mode	ITU-A

The block of coarse time offset estimation using a short preamble is important not only to the ranging of vehicle nodes but also the data communication. Only if the coarse time offset is relatively accurate can the following blocks be correct. In this article we just pay attention to the time offset estimation method using the short preamble in IEEE 802.11p.

#### 5.1.2. Transmission Channel

In an actual application environment, the sent signals may be scattered, reflected, or diffracted by surrounding objects, which may cause some different paths and attenuation. The International Telecommunications Union (ITU) vehicular test environment [24] is a well-known channel model, which can be used to model the multipath delays and channel gains. There are two different delay spreads specified, that is, low delay spread (A), and medium delay spread (B) which are shown in Table 2. The simulation in this paper uses the Channel A.

**Table 2:** ITU’s vehicular test environment parameters.

Tap	Channel A		Channel B		Doppler
	Relative Delay (ns)	Average Power (dB)	Relative Delay (ns)	Average Power (dB)	Spectrum
1	0	0.0	0	–2.5	Classic
2	310	–1.0	300	0	Classic
3	710	–9.0	8900	–12.8	Classic
4	1090	–10.0	12,900	–10.0	Classic
5	1730	–15.0	17,100	–25.2	Classic
6	2510	–20.0	20,000	–16.0	Classic

#### 5.1.3. Performance Metric

In this section, the timing estimation performances are examined in the ITU Channel A parameters. The performance metrics are mean absolute errors (MAE) and the correct percentage of timing estimation error = 0:

(19) $$MAE=\frac{1}{K}\overset{K}{\underset{n=1}{\sum }}\left|D_{n}-{\hat{D}}_{n}\right|$$

where *D_n_* is the peak index corresponding to the *n*th actual time delay, ${\hat{D}}_{n}$ is the peak index corresponding to the *n*th estimated time delay, and *K* is the number of simulation iterations. 

### 5.2. Performance Results and Analysis

The performance in ITU channel was simulated *K* = 2000 times for values of *E*_b_/*N*_0_ from −15 dB to 20 dB. In the remainder of this paper, six methods are considered, those are the CSS method with dynamic threshold (denoted by CSS-dynamic), the CSS method with fixed threshold = 1 (denoted by CSS-fixed), the MathWorks method, the SC-80 method, the RMB method, and the Liu *et al*. method (denoted by Liu). 

#### 5.2.1. Estimation Error

**Figure 13:**
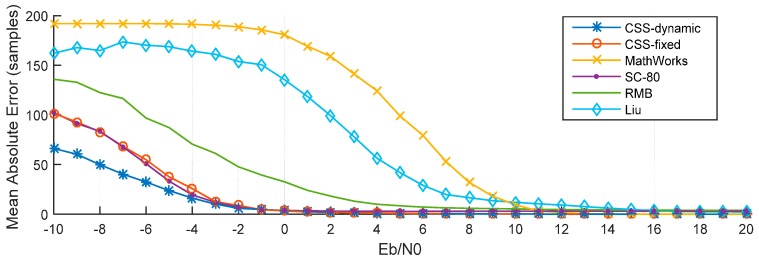
MAE of different methods.

Figure 13 presents MAE for the six methods. These results show that the CSS-dynamic method provides the best performance, particularly at low *E*_b_/*N*_0_ values. 

**Figure 14:**
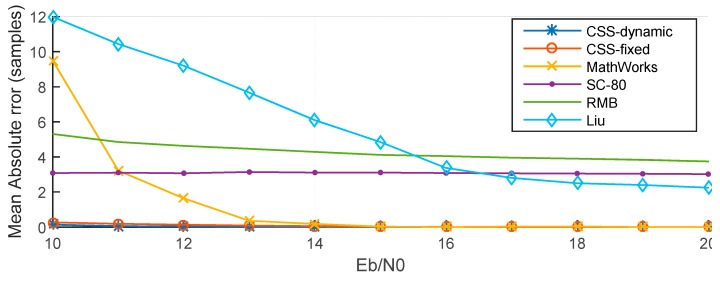
MAE of different methods with E_b_/N_0_ > 10 dB.

For example, when *E*_b_/*N*_0_ = −10 dB, the MAE of the CSS-dynamic method is 66 samples, while MAE are only 101 samples for the CSS-fixed method, 102 samples for the SC-80 method, 136 samples for the RMB method, 162 samples for the Liu method, and 192 samples for the MathWorks method. The MAE of the MathWorks method is the highest when *E*_b_/*N*_0_ is lower than 9 dB. When *E*_b_/*N*_0_ is less than −2 dB the CSS-fixed method is almost the same as the SC-80 method. As shown in Figure 14, because of the multipath, when *E*_b_/*N*_0_ is greater than 13 dB, the MAEs of the CSS-dynamic, CSS-fixed, and MathWorks methods are close to 0. Nevertheless, the MAE of the SC-80 method is close to three samples and the MAE of RMB is close to four samples. For the Liu method when *E*_b_/*N*_0_ is greater than 18 dB, so its MAEs drop down to two samples.

#### 5.2.2. Correct Percentage

**Figure 15:**
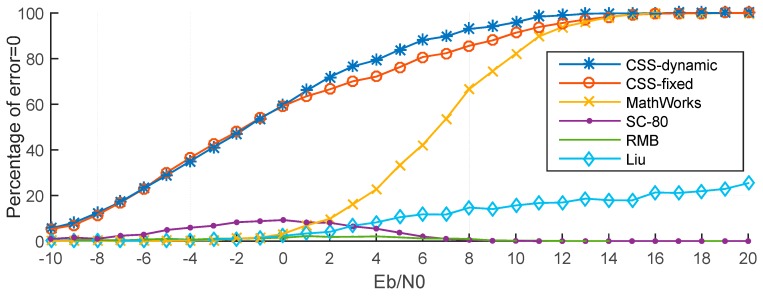
Percentage that the timing error is zero in ITU channel.

**Figure 16:**
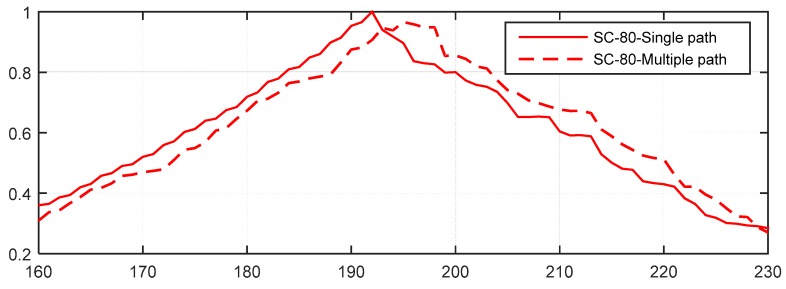
The effect of multipath channel on the timing metric of the SC-80 method.

**Figure 17:**
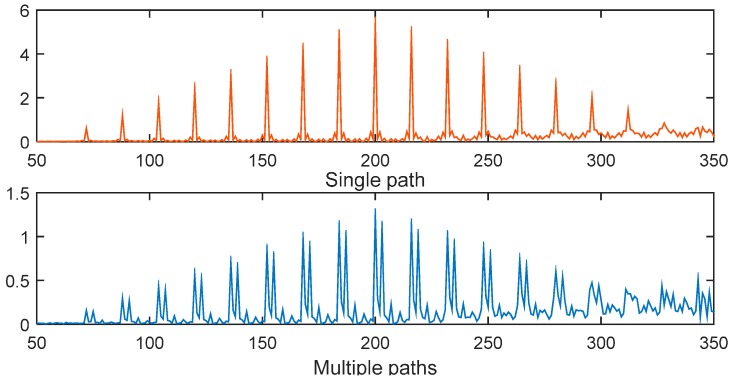
The effect of multipath channel on the timing metric of the CSS method.

Figure 15 presents the percentage of timing estimation error = 0 for the six methods. These results also show that the CSS-dynamic and CSS-fixed methods provide the best performance. For example, when *E*_b_/*N*_0_ = 0 dB, the percentage of the CSS-dynamic and CSS-fixed methods having no error are nearly 59%, whereas for the same percentage with MathWorks, *E*_b_/*N*_0_ should exceed 8 dB, but for the RMB, Liu and SC-80 methods the percentages are too low for all values of *E*_b_/*N*_0_. When *E*_b_/*N*_0_ is greater than 14 dB, the percentages of the CSS-dynamic, CSS-fixed and MathWorks methods are close to 100%, but the others are very low which can also be explained using Figure 14. This is because when *E*_b_/*N*_0_ is greater than 14 dB the MAE of the other three methods are not close to 0 samples. At the same time, for the methods of SC-80 and RMB, the percentage increases at first as *E*_b_/*N*_0_ increases, and then decreases to three or four samples. The reason for the behavior can be explained using Figure 16 and Figure 17 which show the performance in a channel with no noise.

(1) When *E*_b_/*N*_0_ is high, the multipath in the ITU channel causes the peaks to move to the right, making the timing estimation incorrect. Take the SC-80 method as an example, which is illustrated in Figure 16. The percentage will be close to 0 but will not reach 0. On the other hand, Figure 17 gives the corresponding results for the CSS method. This shows that the maximum peak with the CSS method does not move in the multipath channel, and only some smaller peaks are generated, so the percentage will be nearly equal to 100% for a high *E*_b_/*N*_0_.
(2) When *E*_b_/*N*_0_ is very low (close to −10 dB), the noise levels are too high, so it is too difficult to locate the peak, so the percentage is again close to 0.
(3) For *E*_b_/*N*_0_ around 0 dB, the signal energy is close to that of the noise, so it is easier to locate the peak. Now because of the randomization of noise, it’s likely that the estimated time happens to be the true time delayed.

## 6. Conclusions

In this paper, a high precise ranging estimation method based-on the short preamble has been developed for vehicular node positioning using the IEEE802.11p standard. The correlation-summing-skewness analysis method with a dynamic threshold was developed for timing estimation which may be a valuable reference for the study of positioning in vehicular wireless networks. The performance of the CSS-dynamic method is shown to be the best among several well-known methods such as the SC method, MathWorks method, and RMB method, especially in low SNRs, and multi-path environments which is very important for vehicular wireless networks. 

The CSS method can also be used in other wireless communications systems which use OFDM with repeated preamble symbols, such as IEEE 802.11a. On the basis of this coarse time estimation method, we will study on how to use this method in the following fine timing estimation and frequency shift estimation and then the high ranging or position method can be used in the application of cooperative collision avoidance to reduce traffic accidents.
